# Bioinformatics identification and validation of biomarkers and infiltrating immune cells in endometriosis

**DOI:** 10.3389/fimmu.2022.944683

**Published:** 2022-11-29

**Authors:** Hong Jiang, Xia Zhang, Yalan Wu, Baozhu Zhang, Juanbing Wei, Jianhua Li, Yuxiu Huang, Lihong Chen, Xinqin He

**Affiliations:** ^1^ Department of Obstetrics and Gynecology, The First Affiliated Hospital of Fujian Medical University, Fuzhou, Fujian, China; ^2^ Department of Obstetrics and Gynecology, Fu’an Hospital, Fuan, Fujian, China

**Keywords:** endometriosis, immune cell, WGCNA, machine learning, biomarker

## Abstract

**Background:**

Endometriosis (EM) is a common gynecological disorder that often leads to irregular menstruation and infertility. The pathogenesis of EM remains unclear and delays in diagnosis are common. Thus, it is urgent to explore potential biomarkers and underlying molecular mechanisms for EM diagnosis and therapies.

**Methods:**

Three EM-related datasets (GSE11691, GSE25628, and GSE86534) were downloaded from the Gene Expression Omnibus (GEO) which were integrated into a combined dataset after removing batch effect. Differentially expressed immune cell-related genes were obtained by CIBERSORT, WGCNA, and the identification of differentially expressed genes. Random forest model (RF), support vector machine model (SVM), and generalized linear model (GLM) were then constructed and the biomarkers for EM were determined. A nomogram evaluating the risk of disease was constructed and the validity was assessed by the calibration curve, DCA curve, and clinical impact curve. Single-gene Gene Set Enrichment Analysis (GSEA)was performed to explore the molecular mechanisms of biomarkers. The ceRNA regulatory network of biomarkers was created by Cytoscape and potential target drugs were obtained in the DGIdb database (Drug-Gene Interaction database).The expression levels of biomarkers from clinical samples was quantified by RT-qPCR.

**Results:**

The ratio of eight immune cells was significantly different between the eutopic and ectopic endometrium samples. A total of eight differentially expressed immune cell-related genes were investigated. The SVM model was a relatively suitable model for the prediction of EM and five genes (CXCL12, PDGFRL, AGTR1, PTGER3, and S1PR1) were selected from the model as biomarkers. The calibration curve, DCA curve, and clinical impact curve indicated that the nomogram based on the five biomarkers had a robust ability to predict disease. Single gene GSEA result suggested that all five biomarkers were involved in labyrinthine layer morphogenesis and transmembrane transport-related biological processes in EM. A ceRNA regulatory network containing 184 nodes and 251 edges was constructed. Seven drugs targeting CXCL12, 49 drugs targeting AGTR1, 16 drugs targeting PTGER3, and 21 drugs targeting S1PR1 were extracted as potential drugs for EM therapy. Finally, the expression of PDGFRL and S1PR1 in clinical samples was validated by RT-qPCR, which was consistent with the result of public database.

**Conclusions:**

In summary, we identified five biomarkers (CXCL12, PDGFRL, AGTR1, PTGER3, and S1PR1) and constructed diagnostic model, furthermore predicted the potential therapeutic drugs for EM. Collectively, these findings provide new insights into EM diagnosis and treatment.

## Introduction

Endometriosis (EM) is a common chronic gynecological disorder affecting 10%-15% of women of reproductive age (1–3). Patients with EM have clinical features such as dysmenorrhea, chronic pelvic pain, and infertility, which seriously affect the physical and mental health of women of childbearing age (4). Due to the unclear pathogenesis, lack of specific symptoms and non-invasive detection indicators, the diagnosis of endometriosis is often delayed significantly (5). Currently, laparoscopy combined with histopathological examination is the gold standard for the diagnosis of EMs, but laparoscopic surgery carries risks such as trauma, adhesions, and decreased fertility (6, 7). According to statistics, the average interval from the appearance of endometriosis symptoms to the diagnosis is 6.7 years (8), and some even take up to 12 years to diagnose and take corresponding treatment measures (9), resulting in missing the best time for treatment. Therefore, exploring the underlying mechanism of endometriosis and finding effective and non-invasive biomarkers are urgent clinical problems.

The pathogenesis of EM has not been fully understood (10, 11). In recent years, with the deepening of immunological research, it has been found that the cellular and humoral immune functions of EM patients have obvious changes, such as decreased NK cell activity, increased macrophage number and cytotoxicity, abnormal activation of T cells and B cells, and a series of abnormal changes such as the increase of immune inflammatory factors and the production of autoantibodies, so it is speculated that immune factors are one of the important reasons for the occurrence of EM (12–14). However, in the relevant literature on EM, the underlying genes and mechanisms of action related to immune cells are not well studied.

In this study, we downloaded three datasets (GSE11691, GSE25628, and GSE86534) from the Gene Expression Omnibus (GEO, http://www.ncbi.nlm.nih.gov/geo/) to explore potential biomarkers. We employed in-depth bioinformatics analysis to identify EM-related biomarkers, diagnostic models, and possible therapeutic agents. Collectively, these findings provide new insights into EM diagnosis and treatment, and the underlying molecular mechanisms.

## Materials and methods

### Data source

GSE11691 (15), GSE25628 (16), and GSE86534 (17) datasets were extracted and downloaded from Gene Expression Omnibus (GEO) database (https://www.ncbi.nlm.nih.gov/geo/). GSE11691 dataset contains the transcriptome sequencing data of nine eutopic (EU) and nine ectopic (EC) endometrium samples from nine individual women. GSE25628 dataset includes the transcriptome sequencing data of nine eutopic and seven ectopic endometrium samples. GSE85634 dataset comprises the transcriptome sequencing data of four eutopic and four ectopic endometrium samples from four individual women. Immune-related genes (IRGs) were acquired from the Immport database (https://www.immport.org/shared/home).

### Data preprocessing

To combine the transcriptomic data from the above three datasets, we first performed the removal of batch effects by ‘Combat’ in the ‘sva’ R package (18, 19). The ComBat method was employed to normalize the expression values from different batches or platforms. Principal component analysis (PCA) was used to assess whether the batch effect was eliminated.

### Immune cell infiltration analysis

The relative abundance of 22 lymphocyte subtypes in each EC and EU sample were calculated based on the CIBERSORT algorithm and immune cell LM22 gene set (20). After excluding samples with statistically insignificant results (*p* value > 0.05), the Wilcoxon test was used to compare the differences in 22 immune cells between EU and EC samples.

### Weighted gene co-expression network analysis

The transcriptome data of EU and EC samples were incorporated into WGCNA analysis using the R package ‘WGCNA’ (version 1.70-3) (21). The ‘goodSamplesGenes’ function was used to perform sample clustering to identify and remove outliers. For making the co-expression network contented the distribution of scale-free network, a soft-thresholding power was computed with the pickSoftThreshold function. The dynamic tree cutting method was used to identify different modules, with the minimum number of genes in each module of 30. Then, we set the mergeCutHeight as 0.3 to merge similar modules. The correlation between ultimate modules and differential immune cells was further analyzed. The module with the highest correlation with differential immune cells was extracted (|correlation coefficient| > 0.5, *p* value< 0.05), in which genes with |MM|>0.8 and |GS|>0.4 were identified as key module genes. MM represented the correlation of the gene in the module with the module and GS denoted the correlation of the gene with the trait.

### Identification of differentially expressed genes and functional enrichment

Filtering of DEGs between EU and EC samples was performed in the ‘limma’ package (version 3.46.0) (22, 23). The threshold was |log_2_FoldChange (FC)| > 1 and adjusted *p* value < 0.05. The ComplexHeatmap package (version 2.4.3) was utilized to plot the heatmap of the DEGs. Kyoto Encyclopedia of Genes and Genomes (KEGG) pathway analysis was performed by ‘clusterProfiler’ package (24). The threshold for enrichment significance was adjusted *p* value <0.05.

### Screening biomarkers by machine learning models

First, we got the differentially expressed immune cell-related genes by taking the intersection of key module genes, DEGs, and IRGs. Based on differentially expressed immune cell-related genes, the random forest model (RF), support vector machine model (SVM), and generalized linear model (GLM) were constructed by using the ‘caret’ R package (version 6.0-86) (25). The differentially expressed immune cell-related genes regarded as explanatory variables, and EC endometrium samples were served as response variables (26). The three models were then analyzed using the ‘explain’ function in the ‘DALEX’ R package and the cumulative residual distribution was plotted to get the best model. Finally, we analyzed the importance of the variables (differentially expressed immune cell-related genes) in predicting the response variables (EU or EC).

### Construction and validation of a nomogram for endometriosis diagnosis

Subsequently, the ‘RMS’ package (version 6.2-0) was applied to create a nomogram based on the biomarkers, which facilitated the clinical evaluation of the occurrence of endometriosis. Then calibration curve was drawn to assess the predictive accuracy of the nomogram. In addition, the decision curve and clinical impact curve were plotted to evaluate the clinical value of the nomogram.

### Single-gene gene set enrichment analysis

To explore the potential function of biomarkers, we performed Gene Set Enrichment Analysis (GSEA) for single-gene based on GO (‘c5.go.bp.v7.2.symbols.gmt’, ‘c5.go.cc.v7.2.symbols.gmt’, and ‘c5.go.mf.v7.2.symbols.gmt’) and KEGG (‘c2.cp.kegg.v7.2.symbols.gmt’) gene sets in the GSEA software (version3.0). GO enrichment analysis mainly described the biological processes (BP), cellular components (CC), and molecular functions (MF) correlated with genes. According to the expression value of each gene, the correlation coefficients of each gene with all genes in the gene sets were ranked. The threshold for enrichment significance was NOM *p* value < 0.05.

### CeRNA network construction

The ENCORI database (https://starbase.sysu.edu.cn/, screening criteria: CLIP-DATA ≥ 1) and miRWalk database (http://mirwalk.umm.uni-heidelberg.de/, screening criteria: score ≥ 0.95) were employed to predict the target miRNAs of biomarkers. Then the ENCORI database (screening criteria: clipExpNum > 1, degraExpNum > 1) was used to predict lncRNAs targeting the miRNAs. The lncRNA-miRNA-mRNA network was constructed using Cytoscape (version 3.9.0).

### Prediction of potential drugs

Based on the biomarkers of endometriosis, the DGIdb database (https://www.dgidb.org/ search interactions) was utilized to predict potential drugs for the treatment of endometriosis. The network of biomarker-compound pairs was visualized using Cytoscape software (version 3.9.0).

### Sample sources for RT-qPCR validation

The experimental group involved eight patients (Ectopic endometriotic lesion, n=8; Matched Eutopic endometrium, n=8) who attended to the Obstetrics and Gynecology department of the First Affiliated Hospital of Fujian Medical University and underwent hysteroscopy-laparoscopic surgery between December 2021 and January 2022. The patients ranged in age from 28 to 45 years old, with an average age of (31.87 ± 4.28). The experimental group was confirmed the ovarian endometriosis cyst according to the clear intraoperative diagnosis and postoperative pathological examination. The control group involved eight individuals with tubal infertility and underwent hysteroscopy-laparoscopic surgery at the same period (Normal endometrium, n=8). According to intraoperative diagnostic and postoperative pathology, endometriosis, uterine fibroids, uterine polyps, endometrial cancer, and other disorders were not detected among them. These patients ranged of 31 to 50 years old, with an average age of (32.67 ± 2.14). There was no statistically significant age difference between the two groups (P>0.05). To assure the experiment’s correctness, none of the patients had used any hormone medicines in the three months preceding surgery.

This study has been approved by the Ethics Committee of the First Affiliated Hospital of Fujian Medical University, and a notice has been signed with the patients and their families. Informed consent, ethics approval number was [2021 (484)]. The specimens were placed in liquid nitrogen for quick freezing within 30 minutes after *ex vivo*, and then transferred to a - 80°C degree freezer for storage.

### RNA extraction and RT-qPCR

Total RNA from 8 normal endometrium samples, 8 EU samples and 8 EC samples was isolated using Nuclezol LS RNA Isolation Reagent following the manufacturer’s instructions (ABP Biosciences Inc.). Next, total RNA was reverse transcribed into cDNA utilizing the SureScript-First-strand-cDNA-synthesis-kit (GeneCopoeia), according to the manufacturer’s protocol. qPCR was subsequently performed using the BlazeTaq™ SYBR ^®^ Green qPCR Mix 2.0 (GeneCopoeia). The following thermocycling conditions were used for qPCR: 1 cycle at 95°C for 30 sec (initial denaturation), followed by 40 cycles of 10 sec at 95°C (denaturation), 20 sec at 60°C (annealing), and 30 sec at 72°C (extension). The sequences of the primers were listed in **Table 1**. The relative expression level was normalized to the endogenous control GAPDH and calculated using the 2^−ΔΔCq^ method (27). The student’s *t*-test was used to compare the differences between the two groups. The two-tailed *P*-value < 0.05 in statistical analysis was defined as statistically significant.

**Table 1:** The sequences of the primers for qPCR.

Genes	Forword	Reverse
**CXCL12**	AACACTCCAAACTGTGCCCTT	TTTTCCCCACTTTTTCTTCTC
**PDGFRL**	GTCTGGCTGCTGCTTGGT	TGGACGCTTGTTCTTGGG
**AGTR1**	ACGTGACTGTAGAATTGCAGA	GAAAGCCATAAAAAAGAGGAT
**PTGER3**	ACACGGAGAAGCAGAAAGAA	AGGAACACTGGCAGGGTAAG
**S1PR1**	CCACAACGGGAGCAATAACT	GACGATGGAGAGCAGAAGCA
**GAPDH**	CCCATCACCATCTTCCAGG	CATCACGCCACAGTTTCCC

### Statistical analysis

All analyses were conducted using the R programming language, and the data from different groups were compared by the Wilcoxon test. If not specified above, a P-value less than 0.05 was considered statistically significant.

## Results

### Immune cell infiltration between EU and EC samples

**Figure 1:**
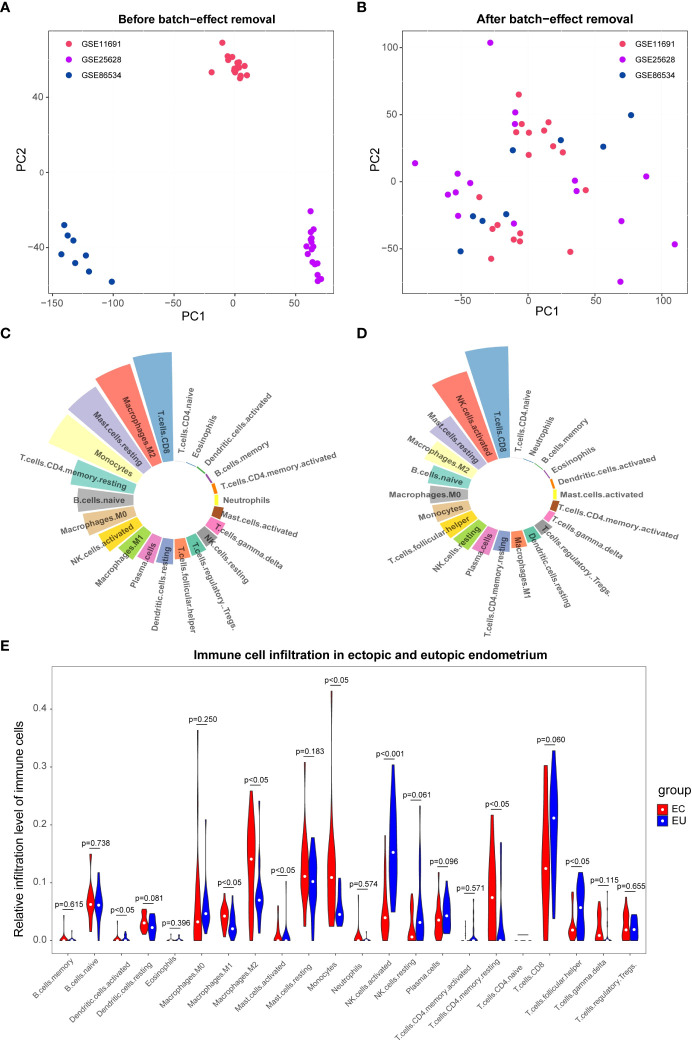
The immune infiltration cell analysis between eutopic endometrium (EU ) and ectopic endometrium(EC). **(A, B)** Principal component analysis (PCA) of the GSE11691, GSE25628, and GSE86534 dataset. The points of the scatter diagrams diaplay the samples based on the top two principal components (PC1 and PC2) of gene expression profiles without **(A)** and with **(B)** the removal of batch effect .The dots in the graph represent samples, and the colors represent corresponding samples different datasets. **(C, D)** Proportion of immune cell infiltration in EU **(C)** and EC **(D)** samples. The different colors represent different immune cells and the size of the loop represents the proportion of immune cells. **(E)** Differences between EU and EC samples for 22 infiltration immune cells. Red represents endometriosis sample (EC), blue represents endometrial eutopic sample (EU).

To remove the batch effect among GSE11691, GSE25628, and GSE86534 datasets, we adopted the ComBat approach. The results of pre- and post-standardized were exhibited in **Figures 1A, B**. Samples from three datasets were clustered according to the top two principal components (PCs) of the unnormalized expression values before the batch effect was removed (**Figure 1A**). In contrast, the scatter-plot based on PCA of normalized expression level manifested that the batch effect arose from different platforms was distinctly removed (**Figure 1B**). The results suggested that cross-platform normalization successfully eliminated the batch effect. We got the normalized expression data of 22 EU and 20 EC samples eventually. Subsequently, we calculated the infiltration proportion of 22 immune cells for each sample using the CIBERSORT algorithm. Two EU samples with non-significant results (GSM296876, p=0.057; GS,629733, p=0.106) were excluded and only 40 samples with *p* value < 0.05 were retained (20 EU and 20 EC samples). **Figures 1C, D** showed the proportion of immune cell infiltration of EU and EC samples respectively. As shown in **Figure 1E**, the proportion of Macrophages M1, Macrophages M2, Monocytes, T cells CD4 memory resting, and Mast cell activated were significantly higher in the EC group compared with the EU group. The proportion of Dendritic cells activated, NK cells activated, and T cells follicular helper were significantly higher in the EU group compared with the EC group. The result indicated that the immune microenvironment of the EC and EU groups was noticeably different.

### Differential immune cells-related genes mined by WGCNA

**Figure 2:**
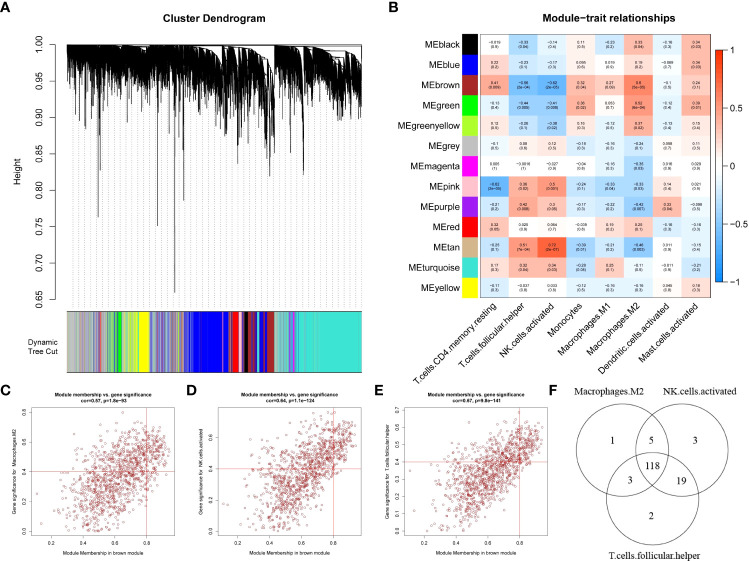
Weighted gene co-expression network analysis based on differential immune infiltration cells. **(A)** Cluster dendrogram. Genes are divided into various modules by hierarchical clustering, and different colors represent different modules, among which gray defaults to genes that cannot be classified into any module. **(B)** The heatmap of modules and immune cells correlation. In the figure, red represents positive correlation, blue represents negative correlation, the darker the color, the stronger the correlation. Each frame labels the correlation coefficient, with the corresponding p-value in brackets. **(C–E)** The scatter plots to show the correlation for MM (X-axis) and GS (Y-axis) in Macrophages.M2 **(E)**, NK.cells.activated **(F)**, and T.cells.follicular.helper (G) for the genes in the brown module. **(F)** Venn diagrams. 118 genes (intersection genes) related to all three types of immune cells were obtained by taking the intersection.

To search for genes associated with differential immune cells, we performed WGCNA based on genes from 20 EC samples and 20 EU samples by using the eight differential immune cells as traits. First, no sample was deleted by sample clustering (**Supplementary Figure 1A**). Next, the soft-thresholding power β = 10 (scale-free R^2^ = 0.85) was selected to construct a scale-free network (**Supplementary Figure 1B**). Then, a cluster dendrogram was constructed and a dynamic tree-cutting was performed (**Figure 2A**). Subsequently, thirteen modules were eventually developed after merging, and the correlations among different modules and differential immune cells was assessed to screened the most significant correlations (**Figure 2B**). Based on the criterion |cor| > 0.5 and p-value < 0.05, the results indicated that the brown module was significantly correlated with T cells folic helper, NK cells activated, and Macrophages M2, was selected as the key module. In the key module, genes with |MM|>0.8 and |GS|>0.4 were detected as key module genes, and totally of 127 key module genes related to Macrophages M2, 145 key module genes related to NK cells activated, and 142 key module genes related to T cells follicular helper were extracted (**Figures 2C–E**). Eventually, we got 118 intersecting genes by overlapping 127 key module genes related to Macrophages M2, 145 key module genes related to NK cells activated, and 142 key module genes related to T cells follicular helper, which were identified as differential immune cell-related genes in endometriosis (**Figure 2F**; **Supplementary Table 1**).

### DEGs between EC and EU samples

The DEGs between EC and EU samples were screened by the ‘limma’ package. The result showed that 542 DEGs, including 294 up-regulated genes and 248 down-regulated genes in EC were identified (**Supplementary Figure 2**, **Supplementary Table 2**). To clarify the KEGG pathway involved by DEGs, we performed a functional enrichment analysis. The result suggested that DEGs were related to multiple pathways such as ‘cell adhesion molecules’, ‘leukocyte transendothelial migration’, ‘renin-angiotensin system’, ‘vascular smooth muscle contraction’, ‘protein digestion and absorption’, ‘axon guidance’, ‘PPAR signaling pathway’, ‘tight junction’, ‘tyrosine metabolism’, ‘steroid hormone biosynthesis’, ‘calcium signaling pathway’, ‘fluid shear stress and atherosclerosis’, ‘focal adhesion’, ‘complement and coagulation cascades’, ‘renin secretion histidine metabolism’, ‘p53 signaling pathway’, and ‘apoptosis’ (**Supplementary Table 3**). Then we predicted the interactions between the DEGs in the STRING database (https://string-db.org/) and constructed a gene-pathway network based on the interaction relationship and KEGG pathways (**Supplementary Figure 3**).

### Biomarkers identified by machine learning models

**Figure 3:**
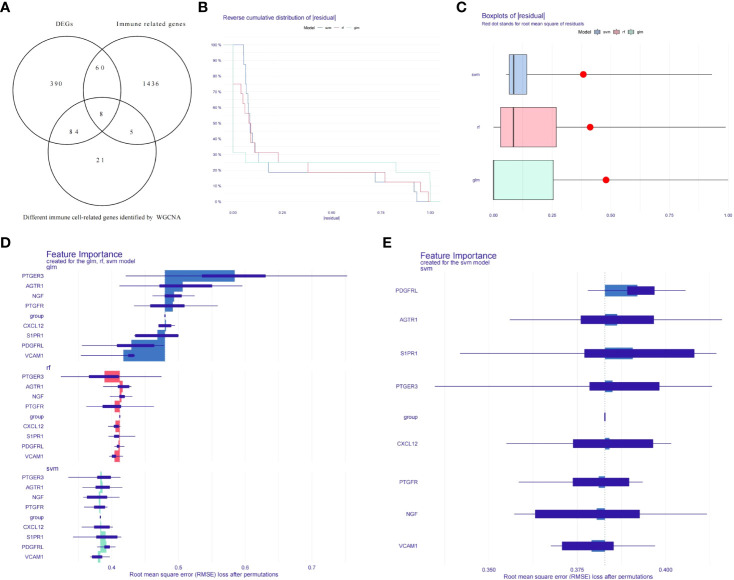
Establishment of diagnostic models and identification of potential diagnostic biomarkers. **(A)** Venn diagrams for differentially expressed immune cell-related genes. **(B)** Cumulative residual distribution plot of sample. **(C)** Boxplot of the residuals of the sample. **(D)** The importance of variables in RF, GLM, and SVM models. **(E)** The importance of variables in SVM models.

To get the differentially expressed levels of immune cell-related genes in endometriosis, we intersected the IRGs, differential immune cell-related genes and DEGs, and eight genes were acquired and enrolled in machine learning analysis (**Figure 3A**). To further narrow the range of key immune cell-related genes, random forest model (RF), support vector machine model (SVM), and generalized linear model (GLM) were created independently on the ground of 20 EC samples and 20 EU samples. Subsequently, the three aforementioned models were then analyzed using the explanatory feature of the “DALEX” package and residual distributions were plotted to obtain the optimal model based on the 20 EC samples and the 20 EU samples. According to the cumulative residual distribution and boxplot, SVM was the best model due to the least sample residual (**Figures 3B, C**). Afterwards, we analyzed the importance of the variables (eight genes) in predicting the response variables (EU or EC) and selected the five most important explanatory variables (CXCL12, PDGFRL, AGTR1, PTGER3, and S1PR1) from the SVM model as biomarkers (**Figures 3D, E**).

### Construction and assessment of a nomogram for endometriosis diagnosis

**Figure 4:**
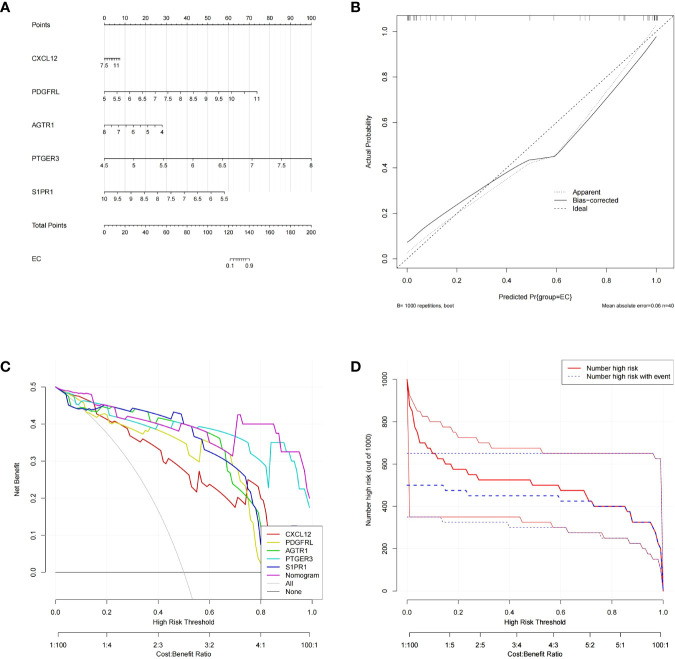
Construction and validation of a nomogram model for EM diagnosis **(A)** The nomogram of diagnostic biomarkers to predict the occurrence of EM. **(B)** The calibration curve to assess the predictive power of the nomogram model.F7 **(C)** The DCA curve to evaluate the clinical application value of nomogram model. **(D)** Clinical impact curves of the nomogram model.

Subsequently, the ‘RMS’ package was applied to establish a nomogram based on the aforementioned five biomarkers (**Figure 4A**). The calibration curve revealed that the predictive accuracy of the nomogram was high as the error between the actual endometriosis risk and the predicted risk was tiny (**Figure 4B**). DCA manifested that the ‘nomogram’ curve was higher than the gray line, ‘CXCL12’ curve, ‘PDGFRL’ curve, ‘AGTR1’ curve, ‘PTGER3’ curve, and ‘S1PR1’ curve, indicated that the patients could benefit from the nomogram at high risk threshold from 0.7 to 1.0 (**Figure 4C**). The clinical impact curve on the ground of the DCA curve was further plotted to evaluate the clinical effects of the nomogram more intuitively. The ‘Number high risk’ curve was close to the ‘Number highDC risk with event’ curve at a high risk threshold from 0.2 to 1, which demonstrated that the nomogram owned powerful predictive ability (**Figure 4D**). These results implied that the five genes may play a key role in the process of endometriosis.

### GSEA for the single gene

**Figure 5:**
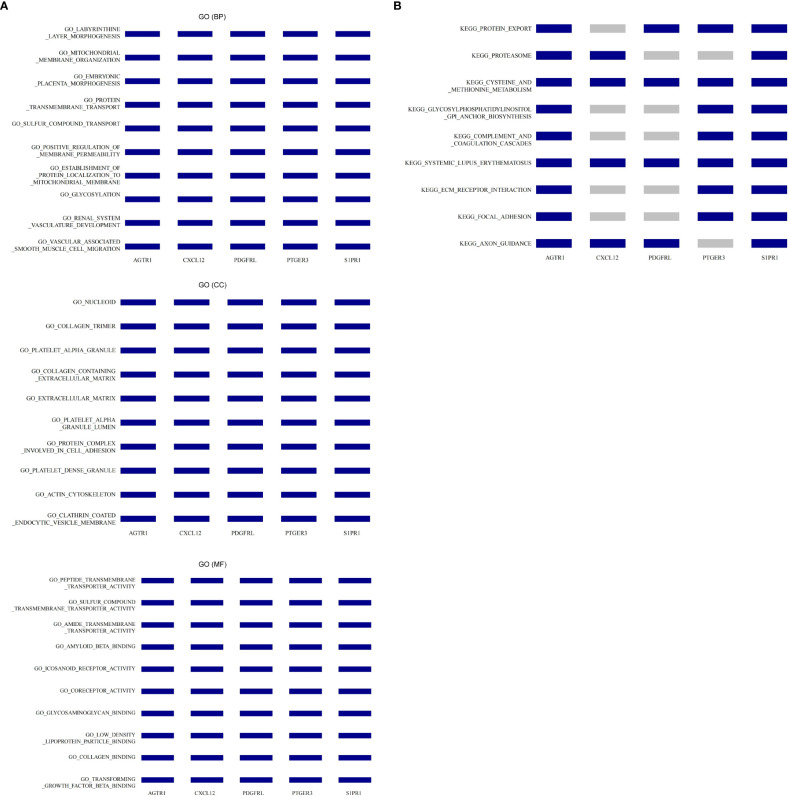
The co-enriched GO entries and KEGG pathways by five diagnostic biomarkers *via* single gene GSEA. **(A)** Co-enriched GO terms in the BP, CC, and MF categories. **(B)** Co-enriched KEGG pathways.

Hence, we conducted GSEA for the single biomarker based on Gene Ontology and KEGG gene sets to explore the potential involved functions and pathways in endometriosis. Top10 items related to all five biomarkers in GO (BP), GO (CC), and GO (MF) category were displayed in **Figure 5A**. As to BP, the five diagnostic genes were all involved in ‘labyrinthine layer morphogenesis’, ‘mitochondrial membrane organization’, ‘embryonic placenta morphogenesis’, ‘protein transmembrane transport’, ‘sulfur compound transport’, ‘positive regulation of membrane permeability’, ‘establishment of protein localization to mitochondrial membrane’, ‘glycosylation’,

‘renal system vasculature development’, and ‘vascular associated smooth muscle cell migration’. As to CC, the five diagnostic genes were all related to ‘nucleoid’, ‘collagen trimer’, ‘platelet alpha granule’, ‘collagen containing extracellular matrix’, ‘extracellular matrix’, ‘platelet alpha granule lumen’, ‘protein complex involved in cell adhesion’, ‘platelet dense granule’, ‘actin cytoskeleton’, and ‘clathrin coated endocytic vesicle membrane’. As to MF, the five diagnostic genes were all associated with ‘peptide transmembrane transporter activity’, ‘sulfur compound transmembrane transporter activity’, ‘amide transmembrane transporter activity’, ‘amyloid beta binding’, ‘icosanoid receptor activity’, ‘coreceptor activity’, ‘glycosaminoglycan binding’, ‘low density lipoprotein particle binding’, ‘collagen binding’, and ‘transforming growth factor beta binding’. Concerning the KEGG pathways, we noted that all five diagnostic genes participated in ‘cysteine and methionine metabolism’ and ‘systemic lupus erythematosus’. AGTR1, PDGFRL, PTGER3, and S1PR1 were involved in ‘protein export’.

AGTR1, CXCL12, PDGFRL, and S1PR1 participated in ‘axon guidance’. AGTR1, PTGER3, and S1PR1were also engaged in ‘glycosylphosphatidylinositol GPI anchor biosynthesis’, ‘complement and coagulation cascades’, ‘ECM receptor interaction’, and ‘focal adhesion’ (**Figure 5B**).

### Diagnostic genes based ceRNA network

To investigate the regulatory mechanisms of biomarkers, we constructed a ceRNA regulatory network. First, we employed the miRWalk and ENCORI database to predict miRNAs associated with biomarkers and obtained 146 mRNA-miRNA pairs. Then, based on the 134 miRNAs acquired above, the related lncRNAs were searched in the ENCORI database and 105 miRNA-lncRNA pairs were achieved. Finally, the ceRNA network containing 184 nodes (5 mRNAs, 134 miRNAs, and 45 lncRNAs) and 251 edges was constructed using Cytoscape software (**Supplementary Figure 4**, **Supplementary Table 4**).

### Potential drugs targeting the diagnostic genes

**Figure 6:**
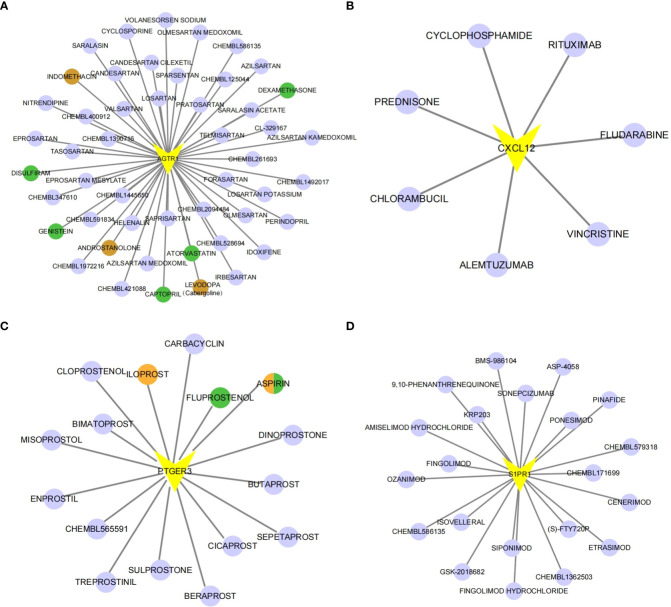
The gene-drug network. The drug-gene network of AGTR1 **(A)**, CXCL12 **(B)**, PTGER3 **(C)** and S1PR1 **(D)**. Drugs marked in brown have already undergone endometriosis-related clinical trials. Drugs marked in green have already been tested on animal models. Orange-colored drugs have already been researched in cell-based experiments.

To explore the potential drugs for endometriosis therapy, we searched the potential drugs targeting the biomarkers in the DGIdb database. As shown in **Figure 6**, seven drugs targeting CXCL12, 49 drugs targeting AGTR1, 16 drugs targeting PTGER3, and 21 drugs targeting S1PR1 were mined. Finally, a lncRNA-miRNA-mRNA-drug network containing 274 nodes (4 mRNA, 45 lncRNAs, 133 miRNAs, and 92 drugs) and 343 edges was generated and exhibited in **Supplementary Figure 5**. In clinical trials, four types of medicines listed above have been shown to be effective in the treatment of endometriosis. In animal studies, seven medicines have been shown to slow the growth of endometriosis. One medicine has been shown to impact the invasive potential of endometrial stromal cells at the cellular level (**Supplementary Table 5**).

### Validation of the expression of diagnostic genes in clinical samples

As shown in **Supplementary Table 2**, CXCL12, PDGFRL, AGTR1, PTGER3, and S1PR1 were up-regulated in EC samples compared to EU samples. To further validate the expression of diagnostic genes, we collected 8 normal endometrium samples, 8 EU samples, and 8 EC samples, extracted RNA, and performed RT-qPCR (**Table 1**). As shown in **Supplementary Figures 6A–E**, the expression of the above five genes was significantly higher in EC samples than in normal endometrium samples, and the differences were significant. We detected that PDGFRL and S1PR1 were significantly highly expressed in EC samples compared to EU samples, which was in line with the result of public database (**Supplementary Figures 6A, B**). Although not statistically significant, we also found a trend of elevated expression of CXCL12 and PTGER3 in EC samples compared to EU samples (**Supplementary Figures 6C, D**). However, there was no significant difference in AGTR1 expression between EC and EU samples (**Supplementary Figure 6E**).

## Discussion

Endometriosis (EM) is a complex systemic gynecological disease (1–3). Although it is a benign disease, it has the characteristics of invasiveness and easy recurrence, which can easily cause long-term chronic pain and infertility in women (4). Due to its increasing incidence, high recurrence rate year by year, and major deficiencies in clinical diagnosis and treatment, EM has been the focus and hotspot of academic research in recent years (5, 6). At present, its pathogenesis is still not fully clear. Studies have shown that the occurrence of the disease may be related to genetic, immune and endocrine factors. Some evidences suggest that abnormal disturbance of immune response and immune dysregulation of ectopic foci are important factors in promoting the growth of ectopic foci, but the in-depth pathogenic mechanism and the molecular mechanism of eutopic endometrial injury are still poorly understood (28). It is necessary to further explore the pathogenesis from the perspective of immunity, so as to provide a new reference for the early, non-invasive diagnosis and treatment of endometriosis.

In the present study, we performed bioinformatics analysis of transcriptome data for eutopic (EU) and ectopic (EC) endometrium samples in the GEO database (GSE11691, GSE25628, and GSE86534 datasets), resulting in the disease-related immune genes, ceRNA network, and non-hormonal drug therapies.

First, an immune infiltration analysis was performed to compare the differences of immune cells between EU and EC samples, and a total of 8 differential immune cells were obtained, which were dendritic cells activated, NK cells activated, monocytes, mast cells activated, macrophages M1, macrophages M2, CD4+T memory resting, and follicular helper T cells (Tfh cells). DCs are antigen-presenting cells that can recognize, process, and present antigenic substances, and can induce the proliferation and differentiation of T lymphocytes (12). In the peritoneal microenvironment of EM patients, immature dendritic cells (iDCs) were significantly increased, while mature dendritic cells (mDCs) were decreased. The inability of iDCs to present ectopic antigens for T cells to recognize leads to a decrease in the clearance rate of ectopic endometrial cells in the abdominal cavity, thereby promoting the occurrence of endometriosis (29). The phenotypic and functional changes of systemic and local NK cells in EM patients have also been reported correspondingly. The mature NK cells in the peritoneal fluid are significantly reduced, and the proportion of immature NK cells is increased (30). Mast cell degranulation can release inflammatory factors. It promotes the persistent occurrence of EM pathological pain, and the number of mast cells and activation in EM lesions is significantly increased (31). In this study, we observed a significant decrease in the abundance of Dendritic cells activated and NK cells activated, and a significant increase in the abundance of mast cells activated in ectopic foci than in eutopic intima, which was consistent with previous studies. Macrophages play an important role in the occurrence and development of endometriosis (32). At different stages of endometriosis development, two different types of macrophages, M1 and M2, are polarized. In this study, the immune infiltration analysis revealed that the content of M1 and M2 macrophages in the ectopic endometrium was higher than that in the eutopic endometrium, which indicated that macrophages play different roles in the occurrence and development of endometriosis at different stages (33). In the early stage of endometriosis, M1 plays the role of releasing inflammatory factors, promoting local inflammatory response, and trying to clear the ectopic endometrium. When endometriosis develops to a certain stage, M2 macrophages increase and are mainly involved in repairing anti-inflammatory, tissue remodeling and pro-fibrotic activities (34). The expression of α-SMA and type I collagen in fibroblasts stimulates their transformation into myofibroblasts; in addition, they can be recruited to the injury site through chemokine-mediated signal transduction pathways such as CCL7 and CCL8, ultimately leading to overproduction of extracellular matrix components, increased collagen deposition, and collagen stabilization and cross-linking to form scar tissue. Fibrosis is the final outcome of patients with endometriosis, which often leads to chronic pain, scarring, infertility, etc. Thus, research on controlling macrophage polarization in order to inhibit or even reverse the epithelial-mesenchymal transition may yield novel ideas for endometriosis therapy (35). T cells also play a role in the immune response to endometriosis. For example, CD4+ resting memory T cells are elevated, indicating that a long-term immune response has been initiated in patients with heterotopic disease. Follicular helper T cells are an independent subset of CD4+ T effector cells. Its most important role is to aid in the formation of the genesis center (GC), the generation of long-lived memory B cells, and the secretion of high-affinity plasma cells (36). The abnormal number or function of Tfh cells and related molecules is related to the occurrence of a variety of autoimmune diseases, such as Sjögren’s syndrome, SLE and Parkinson’s disease. This also shows from one side that endometriosis maybe closely related to autoimmune diseases. If we can deeply understand the role of various immune cells in the formation mechanism of endometriosis, we may find therapeutic targets and provide new directions for the treatment of endometriosis.

The onset of endometriosis is insidious, and the mechanism has not been fully elucidated. At present, there are no sensitive and specific biomarkers for the early diagnosis of endometriosis (9). The gold standard for diagnose endometriosis was invasive procedures such as laparoscopy and histopathology. It leads to delay in diagnosis and treatment of the disease and seriously affects the quality of life of women. Therefore, the search for noninvasive biomarkers is of great significance for the clinical diagnosis and treatment of EM. In order to search for biomarkers related to endometriosis, in the present study, we used random forest (RF), support vector machine model (SVM) and generalized linear model (GLM) to construct three diagnostic models to screen diagnostic genes, and analyzed the diagnostic genes. The model is interpreted, the cumulative residual distribution is calculated, and the SVM model is the best model. The relative importance ranking of different genetic variables for the prediction of the SVM model showed that five variables, CXCL12, PDGFRL, AGTR1, PTGER3 and S1PR1, had a greater impact on the predicted value of the response variable. As a result, we developed a nomogram for endometriosis using these five diagnostic indicators and validated the nomogram using calibration and decision curves. The accuracy of diagnosis is very good. CXCL12 is an important factor in physiological and pathological processes such as embryogenesis, hematopoiesis, angiogenesis and inflammation, and it can activate and induce the migration of hematopoietic progenitor cells, stem cells, endothelial cells and most leukocytes (37). Studies have found that CXCL12/CXCR4/CXCR7 axis mainly promotes the proliferation, migration, and invasion of endometriotic foci through mechanisms such as angiogenesis and induction of stem cell migration In endometriosis, and this process may be regulated by estradiol. Therefore, the CXCL12/CXCR4/CXCR7 axis may become a biomarker of active lesions and a diagnostic indicator of the disease, and may also become a new target for EMT non-hormonal therapy (38). PDGFRL (Platelet-derived growth factor receptor-like), as the protein encoded by it is significantly similar to the extracellular domain of the PDGFR-β subunit, so it is called platelet-derived growth factor receptor-like protein, which is a member of the PDGF family. PDGFRL has been confirmed as a tumor suppressor gene, involved in the regulatory network of PDGF signaling pathway (39). Related studies manifest PDGFRL is related to the steroid hormone feedback mechanism of the reproductive system of chickens, and there are few reports on the relationship with endometriosis. The results of a bioinformatics analysis in humans suggest that PDGFRL in the ectopic endometrium is significantly higher than that in the eutopic endometrium (40). This is consistent with the results of our bioinformatics analysis. This suggests that PDGFRL should play a role in the pathogenesis of endometriosis, and the specific mechanism needs further study. AGTR1 is the receptor for angiotensin II (AngII). Its activation causes a series of biological effects such as vasoconstriction, endothelial dysfunction, platelet activation, activation of platelet-derived growth factor (PDGF) and transforming growth factor beta (TGFβ), causing cell proliferation, and interstitial fibrosis, etc. AGTR1 is highly expressed in epithelial ovarian cancer and is thought to be closely related to the migration, invasion and tumor progression of endometrial cancer cells (41, 42). The research on its relationship with endometriosis is limited and inconclusive. One study found that both the expression level of AGTR1 and the activity of NF-B increased in human endometrial (EM) tissues and stromal cells, and with the activation of AGTR1, the activity of NF-κB also increased, leading the researchers to speculate that high AGTR1 expression increased the uterine Proliferation and invasion of endometrial stromal cells and reduced apoptosis through activation of the NF-κB pathway. This suggests that AGTR1 could be a potential therapeutic target for EM (43).However, in our clinical sample validation, the expression level of ATGR1 in EC was lower than in EU and normal endometrium, which could be attributed to endometriosis heterogeneity and the small sample size. More samples are required for further testing and research.PTGER3 is the strongest affinity receptor for prostaglandin E2 (PGE2), a key intrafollicular mediator of ovulation in many mammalian species. In a comparative laboratory study, PTGER3 and PTGS (prostaglandin endooxidase synthase) were significantly increased in the endometrium of patients with endometriosis compared with those of normal women, and those treated with progesterone had higher levels of PTGS (44). This suggests a role for PTGER3 in the pathogenesis of endometriosis, and why hormone therapy cannot truly cure the disease. S1PR1 is the receptor for sphingosine-1-phosphate (S1P), and S1P is a signaling lipid that plays different biological roles by binding to S1PR1-5. The imbalance of its production and signaling is related to endothelial dysfunction, which is associated with pathological processes such as abnormal angiogenesis (45) Only one study has shown that the activation of the S1P signaling axis mediates the fibrotic effect of TGFβ1 (46). In endometriosis lesions, the high expression of S1P and S1PR1 leads to the migration of endometrial stromal cells, proliferation and angiogenesis, indicating that they play a role in the pathogenesis of endometriosis. The expression of these five genes was significantly higher in ectopic endometrium than in eutopic endometrium, according to the present study. The validation of our clinical sample largely supports this conclusion. However, despite the fact that the expression levels of CXCL12 and PTGER3 in EC samples are higher than those in EU samples, there is no statistical significance, and there is no statistically significant difference between the expression levels of AGTR1 in EC and EU samples, which may be a cause for concern. It may relates to the small size of the validation sample. In order to further validate the expression of AGTR1 in EC and EU and to evaluate the diagnostic value of CXCL12, PTGER3, and AGTR1 in endometriosis, we need to increase the sample size.

In addition to the above-mentioned immune-related genes, our study also focused on the non-coding RNAs associated with the above-mentioned genes (47). Previous research data have shown that non-coding RNAs play an extremely important role in the occurrence and development of endometriosis. The miRNAs are related to cell proliferation, apoptosis, migration, and invasion of endometrial stromal cells, and also have promoting functions such as tissue repair, remodeling and angiogenesis in EM (48). With the in-depth study of lncRNAs in recent years, more and more scholars have found that lncRNAs are abnormally expressed in patients with EM, and participate in the regulation of the occurrence and development of endometriosis by regulating the process of EMT, the transfer of ESCs, and the expression of VEGF. LncRNA can act as a competing endogenous RNA (ceRNA) for miRNA to regulate the proliferation and invasion of ectopic endometrial cells, and play a role in the pathogenesis of endometriosis (49). Our study constructed a ceRNA network, which contained a total of 184 nodes (5 mRNAs, 134 miRNAs, 45 lncRNAs) and 251 edges, which provided a basis for further research on endometriosis.

Currently known drug treatments are mainly hormonal drugs. The hormonal drugs recommended in China include oral contraceptives, high-efficiency progesterone and gonadotropin-releasing hormone agonists (GnRHa). These drugs can inhibit ovarian and induce low estrogen levels. Hormone levels are targeted, leading to side effects such as perimenopausal symptoms, thrombosis, bone loss, etc. Non-hormonal drugs may compensate for the deficiencies of ovarian suppression and progesterone resistance caused by hormonal drug (50).

Through the analysis of the dataset from GEO database, we obtained five immune-related biomarkers for EM, which were potential targets for pharmacological treatment,. Potential drugs for the treatment of endometriosis were predicted using the DGIdb database, and a network of diagnostic marker-molecular compound relationship pairs was visualized with Cytoscape software. Some medications have been utilized clinically within the drug networks we investigated; for example, indomethacin, one of the NSAIDs, is now the drug of choice for endometriosis-related pain (51). Cabergoline, a selective dopamine D2-receptor agonist dopamine receptor agonist, has been shown in clinical trials to inhibit vascular endothelial growth factor and prevent angiogenesis in the progression of endometriosis, thereby shrinking endometriosis lesions (52). As a result, cabergoline has emerged as one of the non-hormonal treatment options for endometriosis. Androstanolone is an androgenic drug. Because of their ability to block estrogen, these drugs have been used to treat endometriosis (53). However, because of androgen side effects such as weight gain, hirsutism, acne, and elevated blood cholesterol levels, is now less commonly used. Low-dose aspirin is thought to improve endometrial progesterone resistance during the window period in endometriosis patients, increasing the expression of the receptivity-related molecule LIF and increasing endometrial receptivity (54). At the same time, it can inhibit the invasiveness of eutopic endometrial stromal cells at the cellular level (55), making it a candidate for endometriosis treatment. At the level of animal experiments, some drugs are thought to have a therapeutic effect on endometriosis. Animal experiments have demonstrated that captopril, atorvastatin, genistein, dexamethasone, disulflram (56–60) have inhibitory effects on endometriosis. Because iloprost (61) and fluprostenol (62) have been shown to promote the progression of endometriosis at the cellular and animal levels, the corresponding inhibitors may be used as a new direction for endometriosis drugs (See **Supplementary excel 1**). Endometriosis is presumed to be an inflammatory disease caused by an immune system imbalance (61); therefore, we boldly hypothesize that selecting drugs from the perspective of immunity and inflammation can provide a new treatment reference for endometriosis (63). Although such drugs have not yet been reported in the field of endometriosis treatment, they can theoretically reduce the body’s immune and inflammatory responses and may be used in the future to treat endometriosis. According to our findings, the use of S1PR1 as a pharmacological target for endometriosis provides a reasonable further application for FTY720 (Fingolmod)-based therapy. The combination of S1PR1 and S1P can affect the migration of T lymphocytes, dendritic cells, and macrophages. FTY720, a structural analog of sphingosine and a functional antagonist of S1PR, is approved by the FDA as a new treatment for multiple sclerosis (64). It is phosphorylated by SphKs *in vivo* to generate phosphorylated FTY720 (p-FTY720), which can act as a ligand for all S1PR1 except S1PR2, thereby strongly inhibiting the outflow of lymphocytes from the thymus and secondary lymphoid organs, reducing the body’s immunity response and inflammatory response (65, 66). Based on this, new ideas can be provided for targeted therapy of endometriosis.

## Conclusions

This study obtained five diagnostic markers for endometriosis, constructed a diagnostic model, explored the roles and related mechanisms of related immune genes and immune cells in endometriosis, and construct a drug network related to endometriosis non-hormonal therapy. The inadequacy is that we have verified by fewer clinical samples, and the results obtained require further experiments and verification of more clinical samples. It provides a new reference for the diagnosis, mechanism research and treatment of the disease.

## Data availability statement

The original contributions presented in the study are included in the article/**Supplementary Materials**. Further inquiries can be directed to the corresponding author.

## Ethics statement

The studies involving human participants were reviewed and approved by Ethics Committee of the First Affiliated Hospital of Fujian Medical University. The patients/participants provided their written informed consent to participate in this study. Written informed consent was obtained from the individual(s) for the publication of any potentially identifiable images or data included in this article.

## Author contributions

HJ, XH, and LC conceived of the presented idea. HJ developed the theory and performed the computations. XZ, YW, BZ, JW, JL, and YW collected samples. XZ and YW performed the real-time quantitative PCR. XH verified the analytical methods. HJ and YW wrote the manuscript with input from all authors. All authors contributed to the article and approved the submitted version.

## Acknowledgments

We thank Gene Expression Omnibus (GEO) database, CIBERSORT database and their contributors for the valuable public datasets used in this study. We thank the sixteen patients with or without endometriosis who underwent surgery at The Affiliated First Hospital of Fujian Medical University in this study. We thank Dr Bin Zhang, Shaozhan Chen, Xiaoli Huang, Xinping Xie for providing samples for us.

## Conflict of interest

The authors declare that the research was conducted in the absence of any commercial or financial relationships that could be construed as a potential conflict of interest.

## Publisher’s note

All claims expressed in this article are solely those of the authors and do not necessarily represent those of their affiliated organizations, or those of the publisher, the editors and the reviewers. Any product that may be evaluated in this article, or claim that may be made by its manufacturer, is not guaranteed or endorsed by the publisher.

## Supplementary material

Supplementary Figure 1 Sample clustering and soft-threshold power screening in the WGCNA. **(A)** Sample clustering was conducted to detect outliers. All samples are located in the clusters and pass the cutoff thresholds. **(B)** Soft-thresholding power analysis was adopted to obtain the scale-free fit index of network topology in the WGCNA.

Supplementary Figure 2 The heatmap showing expression profiles of the DEGs.

Supplementary Figure 3 Gene-KEGG pathway network. The red swallowtail quadrilaterals represent the KEGG pathway, and the blue-purple circles represent DEGs.

Supplementary Figure 4 The ceRNA network. Red dovetail quads represent mRNAs, blue-purple diamonds represent lncRNAs, and yellow-green circles represent miRNAs.

Supplementary Figure 5 The lncRNA-miRNA-mRNA-drug network. Red dovetail quadrilaterals represent mRNAs, blue-purple diamonds represent lncRNAs, yellow-green circles represent miRNAs, and green triangles represent drugs.

Supplementary Figure 6 Validation of the expression of five key genes between inclinical sample by RT-qPCR. * indicates p value < 0.05.
