# Supplementary figures and images for: Bioinformatics identification and validation of biomarkers and infiltrating immune cells in endometriosis
Source: Front Immunol. 2022 Nov 29;13:944683. doi: 10.3389/fimmu.2022.944683 (PMC9745028; doi:10.3389/fimmu.2022.944683)

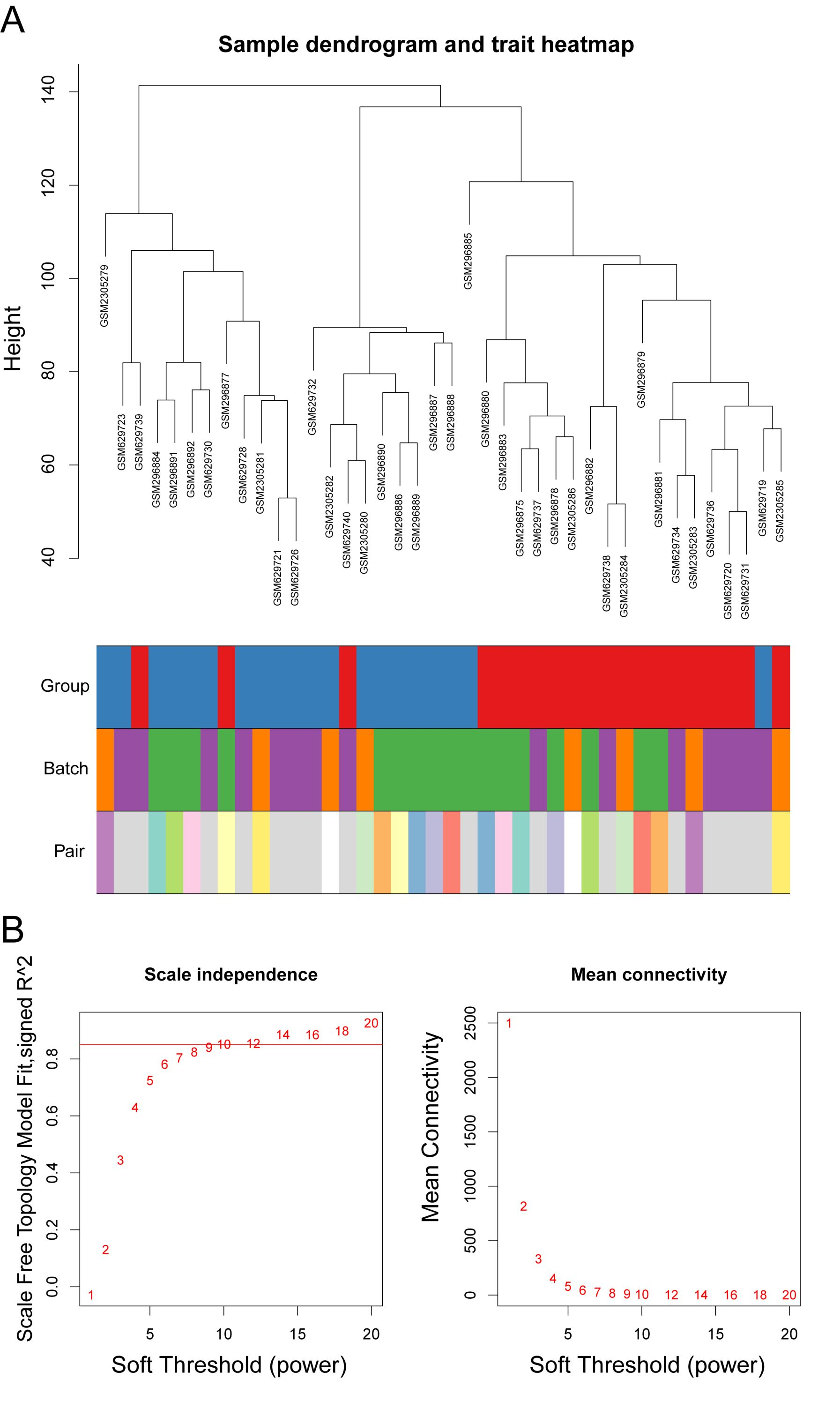

Supplement: Supplementary Figure 1 — Sample clustering and soft-threshold power screening in the WGCNA. (A) Sample clustering was conducted to detect outliers. All samples are located in the clusters and pass the cutoff thresholds. (B) Soft-thresholding power analysis was adopted to obtain the scale-free fit index of network topology in the WGCNA. [file Image_1.tif]

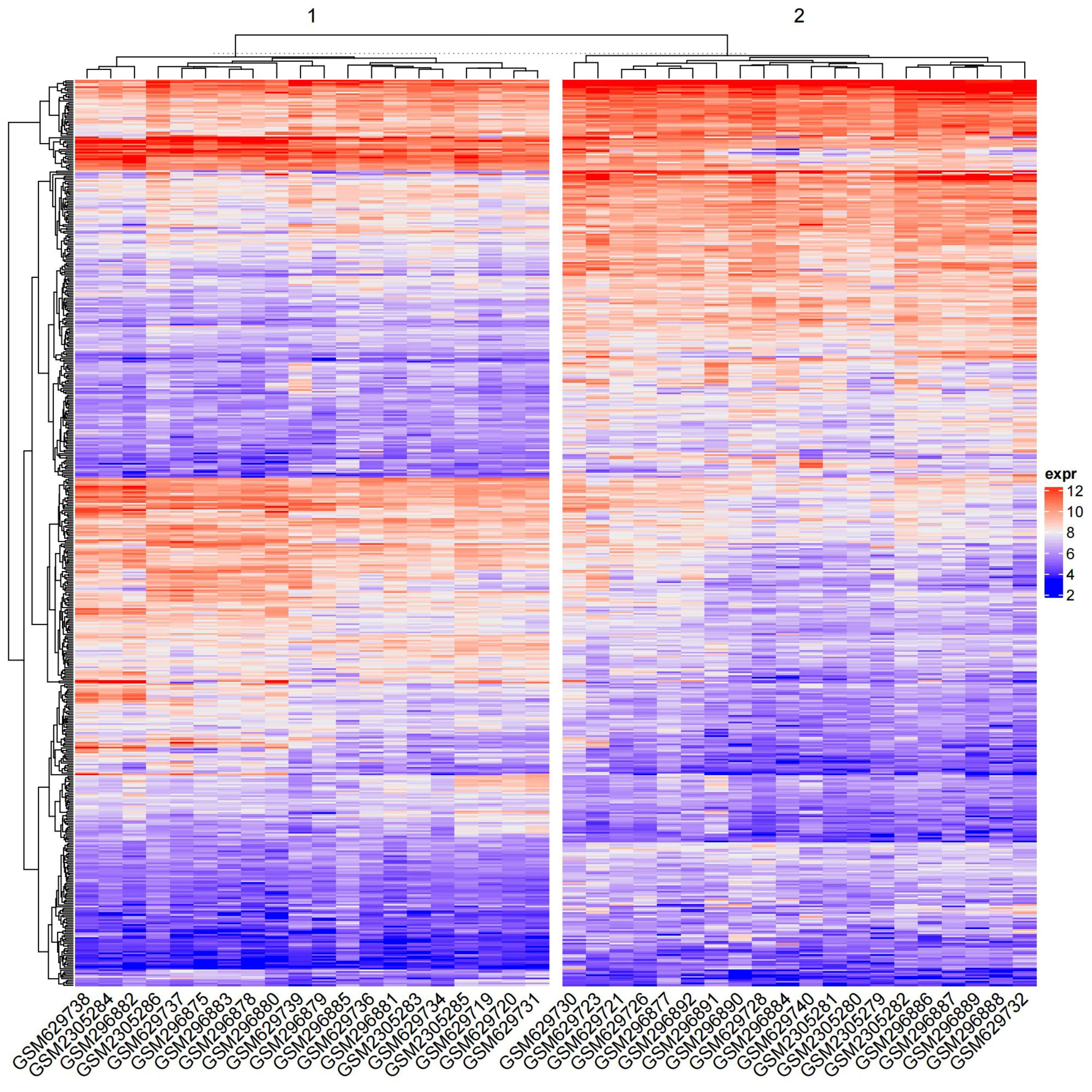

Supplement: Supplementary Figure 2 — The heatmap showing expression profiles of the DEGs. [file Image_2.tif]

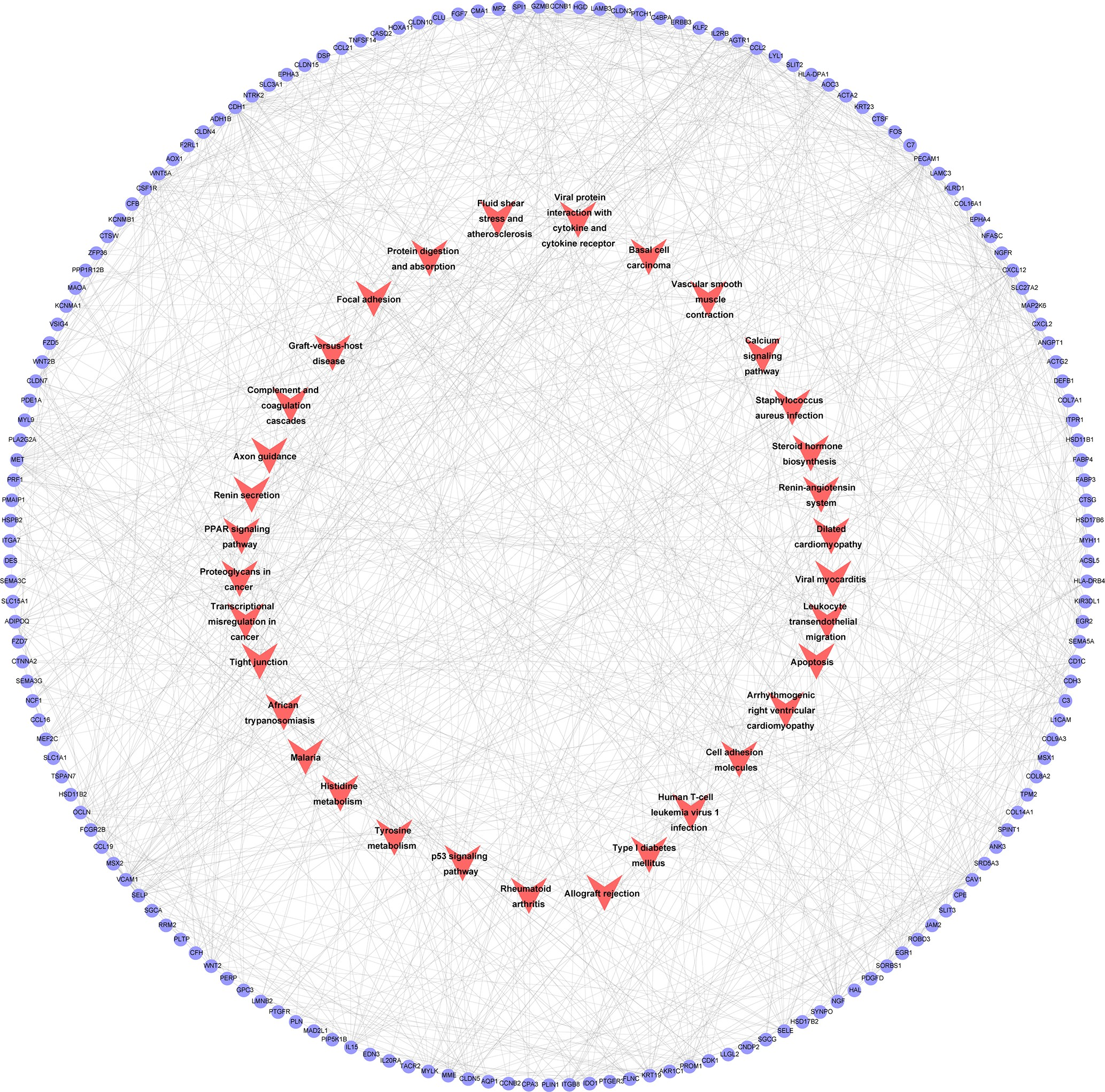

Supplement: Supplementary Figure 3 — Gene-KEGG pathway network. The red swallowtail quadrilaterals represent the KEGG pathway, and the blue-purple circles represent DEGs. [file Image_3.tif]

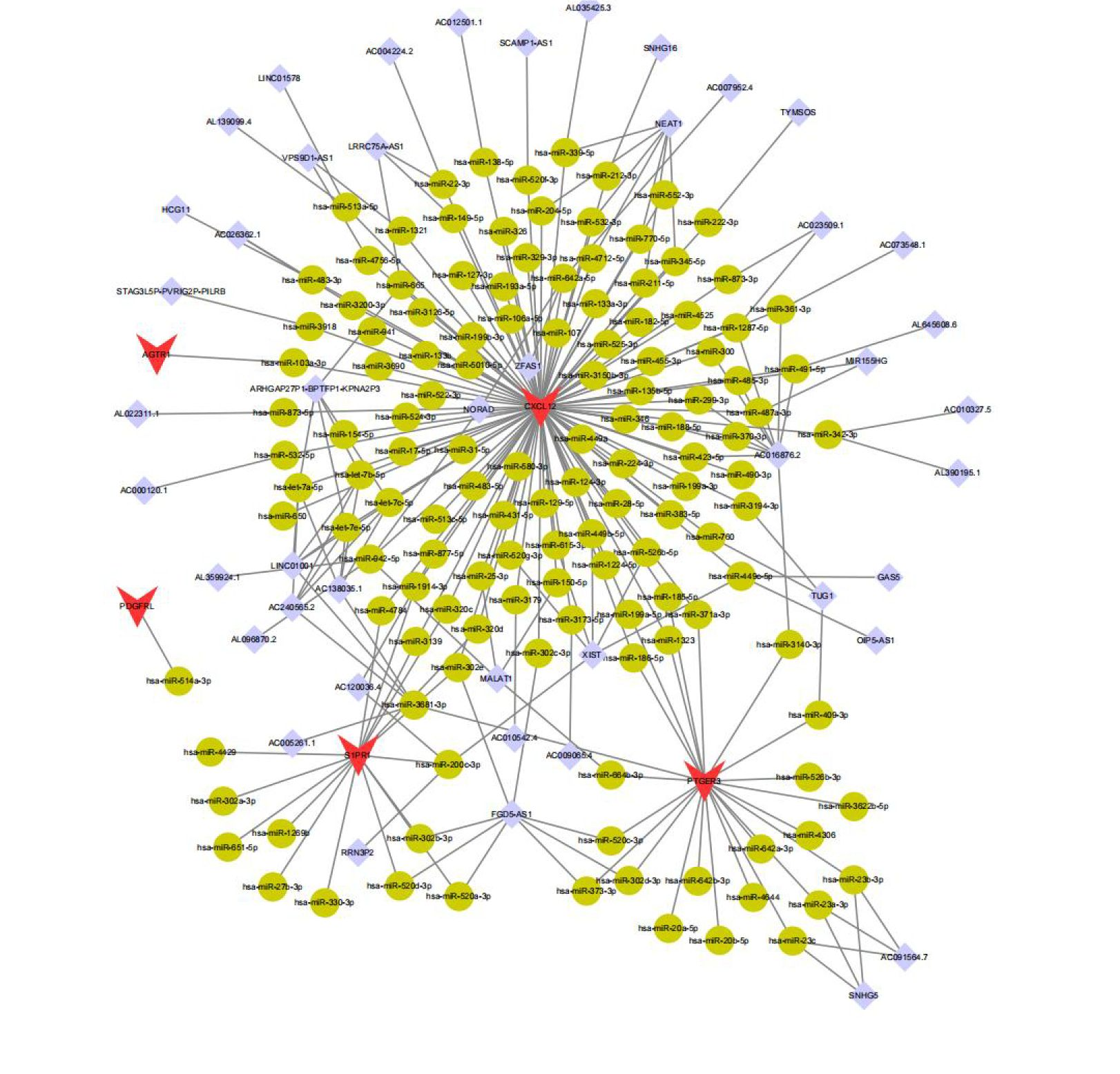

Supplement: Supplementary Figure 4 — The ceRNA network. Red dovetail quads represent mRNAs, blue-purple diamonds represent lncRNAs, and yellow-green circles represent miRNAs. [file Image_4.tif]

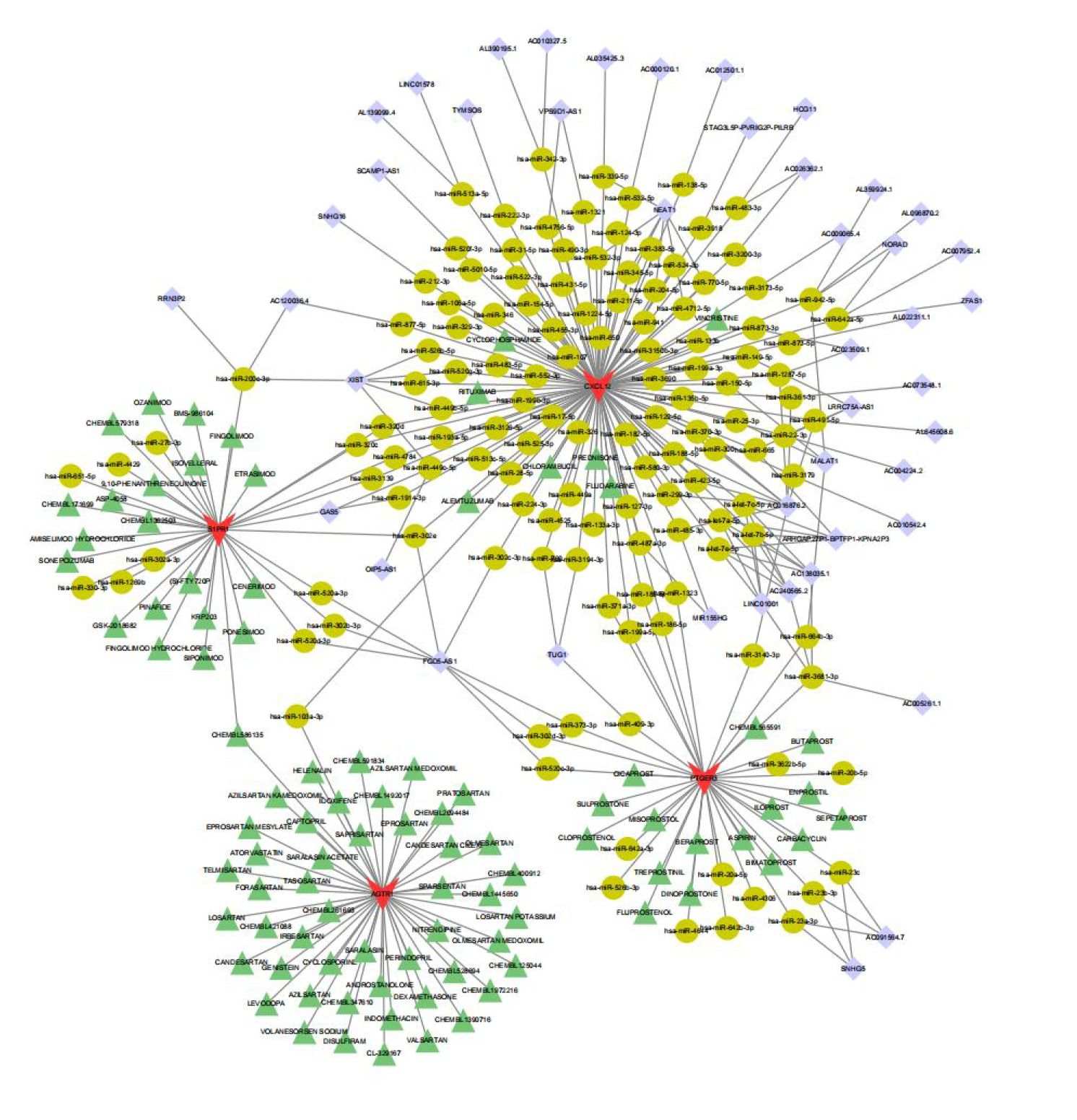

Supplement: Supplementary Figure 5 — The lncRNA-miRNA-mRNA-drug network. Red dovetail quadrilaterals represent mRNAs, blue-purple diamonds represent lncRNAs, yellow-green circles represent miRNAs, and green triangles represent drugs. [file Image_5.tif]

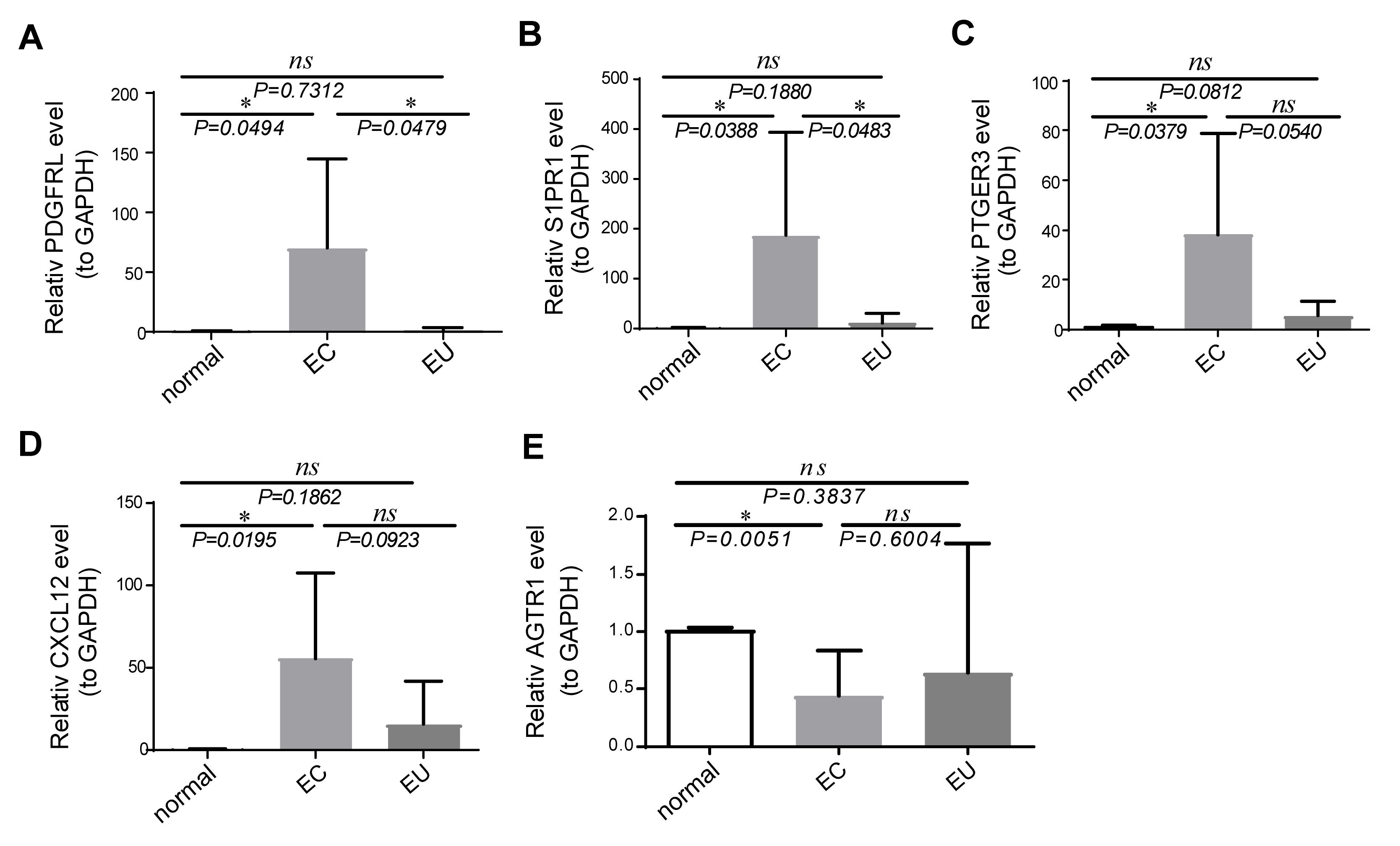

Supplement: Supplementary Figure 6 — Validation of the expression of five key genes between inclinical sample by RT-qPCR. * indicates p value < 0.05. [file Image_6.tif]
